# Bridging the gap in knowledge and use of antibiotics among pediatric caregivers: comparing two educational interventions

**DOI:** 10.1186/s40545-023-00578-5

**Published:** 2023-06-19

**Authors:** Isabel Naomi Aika, Ehijie Enato

**Affiliations:** Department of Clinical Pharmacy and Pharmacy Practice, Faculty of Pharmacy, University of Benin, Benin, Nigeria

**Keywords:** Education, Pediatric, Caregivers, Antibiotics

## Abstract

**Background:**

The inappropriate use of antibiotics in pediatrics contributes to antimicrobial resistance. Behavior change intervention involving education to improve the use of antibiotics is a strategy included in antimicrobial stewardship. This study aims to evaluate and compare the impact of two educational interventions on knowledge of antibiotic and its use among pediatric home caregivers.

**Methods:**

This was a cross-sectional study conducted in the pediatric out-patient department of a healthcare facility. A structured questionnaire was administered to sixty pediatric caregivers. Pediatric caregivers were grouped in two of 30. Caregivers in a group filled the questionnaire, and refilled the same questionnaire after a one-on-one education. The second arm refilled the questionnaire after a group education. Ethical clearance was obtained and participants gave consent. Data analysis was done using SPSS version 22 and Graph pad Instat, *p* values < 0.05 were considered significant.

**Results:**

Fouty-nine (81.7%) participants believe that antibiotics can treat malaria infection [8(13.3%) after education)], 43(71.7%) of respondents agreed that antibiotics can be used to treat all kinds of diarrhea, while 45(65%) of them thought that antibiotics in powder form can be reconstituted with hot or warm water before use [7(11.7%) after education]. Mean score among the sixty participants before and after education on knowledge and use of antibiotics were 36.1 ± 6.467 versus 46.7 ± 4.027 (*p*≤0.0001) and 29.82 ± 4.949 versus 36.92 ± 3.997 (*p*≤ 0.0001), respectively. Mean score on knowledge and use of antibiotics for one-on-one versus group education were 46.7 ± 4.027 versus 43.3 ± 6.249 (*p* = 0.022) and 37.9 ± 3.044 versus 35.93 ± 4.608 (*p* = 0.039), respectively.

**Conclusions:**

Many pediatric caregivers had poor knowledge on antibiotics and use which improved significantly after education. One-on-one education has more impact than group education. Pharmacists and other healthcare professionals can use counseling opportunity to inform caregivers on appropriate knowledge and use of antibiotics consistently to change behavior.

**Supplementary Information:**

The online version contains supplementary material available at 10.1186/s40545-023-00578-5.

## Background

Antimicrobial resistance (AMR) is considered to be a global crisis with three sub-dimensions: a health crisis, an economic crisis, and a security crisis. Children are the most hit of this crisis from antibiotic resistance majorly, because they are prone to infectious diseases. In Nigeria, infectious diseases that are transmissible such as measles, diarrhea, respiratory tract infections and tuberculosis accounted for more than 60% of mortality in 2015, these infections are usually treated with antimicrobials [1]. Children below the age of 14 years constitute more than 40% of the population in Nigeria, while children aged 5 years and below make up the bulk of patients attending the pediatric out-patient clinics in most health care facilities [2, 3].

Antimicrobial stewardship practice remains an effective strategy to reduce the burden of global antimicrobial resistance. One of the strategies employed in antimicrobial stewardship is behavior change interventions (BCIs). BCIs are coordinated sets of activities designed to change specified behavior patterns, the most widely used of these interventions are educational-based [4, 5]. Many studies have shown the effectiveness of educating healthcare professionals and the positive impact on behavioral change in prescribing antibiotics and complying with antimicrobial stewardship practice in various healthcare settings [6–10]. However, the O’Neill report emphasizes the need for AMR awareness interventions directed not just to healthcare professionals, but toward the public and development of a uniform, globally consistent set of AMR messages that could be then tailored to meet the specific demands of local settings [11]. This recommendation is based on the fact that inappropriate use of antibiotics is highly influenced by human behavior at many levels of society. Interventions targeting patients and the wider community, potentially using communication and environmental/social planning policies are required to improve the use of antibiotics and leaving out these key agents could hinder efforts void [12]. Against this background, this study aims to evaluate and compare the impact of two educational interventions on knowledge of antibiotic and use of antibiotics among pediatric home caregivers and to determine the best approach to convey such intervention.

## Methods

### Study design/setting

This was an analytical cross-sectional study conducted in the pediatric out-patient department of Central hospital, a secondary healthcare facility in Benin City, Edo State, located in Southern Nigeria.

### Data collection

A self-administered structured interviewer’s questionnaire was used as data collection tool. The questionnaire was in three sections. Section one provided details of participants demographics, section two was a 10-item question on knowledge of antibiotics in treatment of infections and measures to reduce infection and use of antibiotics in pediatrics, while section three was an 8-item question on appropriate use of antibiotic in pediatric dosage form. Questions were developed from extensive literature review on similar studies that have been done [13–15]. Responses were presented in a 5-likert scale of strongly disagree, disagree, undecided, agree and strongly agree with a score of 1 to 5, respectively. Reverse scoring was done for question 1 to 5 in section two and question 1 to 8 in section three, giving a total score of 50 and 40 for sections two and three, respectively. The knowledge section score was divided in two, 40–50 was considered good score, < 40 was considered poor, while the practice section score was also grouped in two, 32–40 as good score, and < 32 as poor.

### Data collection procedures/protocols

A total of sixty participants were included in the study. Participants were drawn from parents at the pediatric outpatient waiting area to take their wards for physician consultation who have used pediatric antibiotic in the pasts. Participants were grouped in two, 30 caregivers were approached individually and asked to fill questionnaire after consent was sought. A one-on-one educational intervention on appropriate knowledge and use of antibiotics based on the questionnaire was carried out orally by a pharmacist to the individuals after they have filled the questionnaire, they are then given the same questionnaire to refill. Another 30 participants were given a group educational intervention after they filled the questionnaire. The group educational intervention included oral presentation of appropriate knowledge and practice of antibiotics based on the questionnaire together with a poster flip chart presentation to aid visual communication by the same pharmacist. The pharmacist who did the educational intervention is currently an academic pharmacist and a clinical pharmacist with 8 years experience, and prior 4 years combined experience in community and hospital practice. Among the duties in academic practice, the pharmacist serves as a clinical preceptor for the clinical clerkship program for both undergraduate Pharm D and Master students in hospitals and community pharmacies as part of their academic curriculum. The one-on-one session was done in 3 consecutive days of ten participants per day. The group education was done a week after the one-on-one session. The group session was done in a day. In both educational interventions, the content of education was the same and participants were allowed to ask questions for clarity purpose after the education. The one-on-one educational intervention took between 10 and 20 min, while the group intervention took 40 min. Both educational interventions were done in Nigerian Pidgin (an English-based creole language spoken as a lingua franca across Nigeria).

### Data analysis

Data were entered into excel and sorted (Additional file 1). Data analysis was done using SPSS version 22, discrete variables were expressed as percentages and proportions. Graph pad Instat was used to compute means and conduct student t test. Mean scores were compared between groups to identity any difference in educational interventional approach and within study population to determine the impact of educational intervention. Statistical differences were considered significant at *p* value of < 0.05 at 95% Confidence interval.

## Results

A total of 60 caregivers participated in the study. The mean age was 34.10 ± 6.435. More than half of them were aged between 31 and 40. Majority of the participants were married (93.3%), more than half (74.5%) had two of more children and half of them attained post-secondary education (50%) (see Table 1).

**Table 1:** Socio-demographic characteristics of participants (*N* = 60)

Variable	Frequency (*N*)	Percentage (%)
Age		
20–30	18	30
31–40	34	56.7
41–50	8	13.3
Sex		
Female	60	
Male	0	100
Marital status		
Married	56	93.3
Divorced	2	3.3
Separated	1	1.7
Single	1	1.7
Education		
No formal	0	0
Primary	7	11.7
Secondary	23	38.3
Post-secondary	30	50
Employment		
Employed	34	56.7
Unemployed	26	43.3
Income (₦)		
< 30,000	34	56.7
30,000– ≤ 50,000	6	10
> 50,000– < 100,000	6	10
≥ 100,000	4	6.7
None	10	16.7
Number of children		
1	16	26.5
2	14	23.3
3	16	26.7
4	9	15
> 4	5	8.3

Of the 60 participants, 14(23.3%) of them had a good score (between 40 and 50) before education which increased to 49 (81.7%) after education on knowledge of antibiotics and infection prevention, whereas 46 (76.7%) of them scored poorly (> 40) before education, which reduced to 11(18.3%) after education about knowledge of antibiotics and ways to reduce infection in children. Forty-nine (81.7%) [8(13.3%)] after education] participants believed that antibiotics can treat malaria infection, 37(78.3%) [7(11.7%) after education] thought that bacteria is the only cause of infection presenting as cough, catarrh and cold in children, 43(71.7%) [7(11.7%) after education] of respondents either agreed to some extent or are unsure that antibiotics can be used to treat all kinds of diarrhea and dysentery, and 31(51.7%) [10(15.6%) after education] believed that antibiotics do not cause any side effects. Table 2 shows that high mean scores were noted for areas relating to infection prevention. The mean scores increased in all domains (*p* < 0.05) after educational intervention on knowledge of antibiotics and infection prevention compared to before education. The mean score increased from 35.70 ± 5.616 before education to 45 ± 5.487 after education (*p* < 0.0001).

**Table 2:** Comparison of knowledge of antibiotics and infection prevention before and after education (*N* = 60)

Variable	Mean before *M* ± SD	Mean after *M* ± SD	*P* value	Confidence interval	*t*-score
Only Bacteria cause cold, fever, sore throat and catarrh, in infection in children	2.4 ± 1.196	4.3 ± 1.174	< 0.0001	− 2.328–(− )1.539	8.754
Antibiotics can treat all kind of diarrhea and dysentery	2.58 ± 1.094	4.36 ± 1.134	< 0.0001	− 2.164–(− )1.403	9.372
Antibiotics can treat malaria infection	2.41 ± 1.164	4.23 ± 1.198	< 0.0001	− 2.251–(− )1.382	8.365
Antibiotics can be stopped when symptoms of infection disappear even before the required number of days	3.8 ± 1.282	4.48 ± 0.9296	0.0005	− 0.9533–(− )0.2801	3.666
Antibiotics do not have side effects that are serious	3.21 ± 1.263	4.2 ± 1.162	< 0.0001	− 1.334–(− )0.6322	5.604
Complete Immunization of children prevents them from frequent infection	4.1 ± 0.9690	4.7 ± 0.6457	< 0.0001	− 0.8434–(− )0.3566	4.932
Good hand hygiene among children prevents them from frequent infection	4.26 ± 0.8610	4.72 ± 0.4544	0.0002	− 0.6802–(− )0.2198	3.912
Practice of food hygiene prevents them from frequent infection	4.25 ± 0.9677	4.63 ± 0.6630	0.0158	− 0.6919–(− )0.7477	2.486
Clean home and good waste disposal prevent children from frequent infection	4.31 ± 0.8129	4.7 ± 0.4621	0.0017	− 0.6168–(− 00.1499	3.286
Good respiratory hygiene prevents children from frequent infection	4.28 ± 0.7152	4.63 ± 0.6630	0.0071	− 0.6009–(− )0.9912	2.791
	35.7 ± 5.616	45 ± 5.487	< 0.0001	− 11.010–(− 00.7590	10.880

Twenty-three (38.3%) [55 (91.7%) after education] participants scored between 32 and 40, while 37(61.7%) [5(8.3%) after education] scored < 32 on the responses about how they use antibiotics before educational intervention. Sixteen (26.7%) [3 (5%) after education] of respondents asked their doctor or pharmacist to prescribe antibiotics for their child even if they do not want to, 43(71.7%) [4(6.7%) after education] of them thought that antibiotics in powder form can be reconstituted with hot or warm water before use, 19(31.7%) [2(3.4%) after education] of respondents agreed that powder antibiotics that has been reconstituted with water can be stored anywhere, while 21(31.5%) [3(5%) after education] of pediatric caregivers believed that an antibiotic that was previously prescribed before for a child can be bought again without doctor’s consultation for the same child or another child with similar symptom. The mean score increased from 29.82 ± 4.949 before education to 36.92 ± 3.997 after education (*p* < 0.0001) (Table 3).

**Table 3:** Comparison of antibiotics use before and after education (*N* = 60)

Variable	Mean before *M* ± SD	Mean after *M* ± SD	*P* value	Confidence interval	*t*-score
I ask my doctor or pharmacist to prescribe antibiotics for my child even if they do not want to	3.8 ± 1.190	4.6 ± 0.8065	< 0.0001	− 1.177–(− )0.4231	4.247
Antibiotics in powder form can be mix with hot or warm water before use	2.41 ± 1.344	4.54 ± 0.8968	< 0.0001	− 2.568–(− 01.737	10.364
It can be confusing sometimes to know the quantity of water to mix powder antibiotics with	3.9 ± 1.217	4.48 ± 0.9296	0.0004	− 0.8926–(− 00.2740	3.774
Measuring the quantity of antibiotic to give a child from powder antibiotic that has been mix can be difficult	4.11 ± 0.9931	4.55 ± 0.8321	0.0033	− 7162–(− )0.1505	3.066
Powder antibiotics that has been mixed with water can be stored anywhere	3.63 ± 1.164	4.68 ± 0.5964	< 0.0001	− 1.371–(− )0.7295	6.554
Powder antibiotics that has been mixed with water can be reused after some time either for the same child or another child	4.23 ± 1.031	4.6 ± 0.4034	0.0001	− 0.8414–(− )0.2919	4.127
An antibiotic that was previously prescribed before for a child can be bought again without doctor’s consultation for the same child or another child with similar symptom	3.5 ± 1.524	4.56 ± 0.8707	< 0.0001	− 1.447–(− 00.6866	5.615
Antibiotics can be given at any time outside the recommended dosing time	4.21 ± 1.059	4.76 ± 0.5326	< 0.0001	− 0.8080–(− )0.2920	4.266
	29.82 ± 4.949	36.92 ± 3.997	< 0.0001	− 8.432–(− )5.768	10.663

In the one-on-one education arm, before education, 6(20%) participants had good score of between 40 and 50 and 24 (80%) scored poorly (< 40), while 29(96.7%) had good score of between 40 and 50, and 1(3.3%) scored poorly after education on knowledge of antibiotics and infection prevention. The mean score increased from 36.1 ± 6.467 before education to 46.7 ± 4.027 after education (*p* < 0.0001). For antibiotic use session, of the thirty participants, 17(56.5%) had good score (between 32 and 40) but 13(43.3%) scored poorly (< 32) before education, while 28(93.3%) had a good score, with 2(6.7%) scoring poorly after education on antibiotic use. The mean score increased from 31.3 ± 5.318 before education to 37.9 ± 3.044 after education (*p* < 0.0001).

In the group education, 8(26.7%) caregivers had good score (between 40 and 50) with 22 (73.3%) scoring poorly (< 40) before the group education, while 20(66.7%) had good score, and 10(33.3%) scoring poorly after education on knowledge of antibiotics and infection prevention. The mean score increased from 35.3 ± 4.692 before group education to 43.3 ± 6.249 after group education (*p* < 0.0001). For antibiotics use, of the thirty participants, 6(20%) had good score (between 32 and 40) but 24 (80%) scored poorly (< 32) before education, while 27(90%) had good score, with only 3(10%) scoring poorly after education on use of antibiotics. The mean score also increased from 28.33 ± 4.122 before education to 35.9 ± 4.608 after education (*p* < 0.0001). Comparison of the mean score in knowledge of antibiotics and infection prevention between group and one-on-one educational intervention is represented in Table 4. The mean score was higher (46.7 ± 4.027) for one-on-one compared with 43.3 ± 6.249 for the group after educational intervention (*p* = 0.0219).

**Table 4:** Comparison of knowledge of antibiotics and infection prevention between one-on-one and group after education (*N* = 60)

Variable	One-on-One *M* ± SD	Group Mean *M* ± SD	*P* value	Confidence interval	*t*-score
Only Bacteria cause cold, fever, sore throat and catarrh, and infection in children	4.66 ± 0.8023	4 ± 1.390	0.0043	0.0211–1.312	2.112
Antibiotics can treat all kind of diarrhea and dysentery	4.73 ± 0.5208	4 ± 1.438	0.0124	0.1706–1.296	2.665
Antibiotics can treat malaria infection	4.6 ± 0.8137	3.86 ± 1.408	0.0267	0.0908–1.376	2.334
Antibiotics can be stopped when symptoms of infection disappear even before the required number of days	4.66 ± 0.4795	4.3 ± 1.208	0.1331	− 0.1185–0.8519	1.546
Antibiotics do not have side effects that are serious	4.6 ± 0.724	3.8 ± 1.375	0.0137	0.1767–1.423	2.625
Complete Immunization of children prevents them from frequent infection	4.8 ± 0.4068	4.6 ± 0.8137	0.2266	− 0.1311–0.5311	1.235
Good hand hygiene among children prevents them from frequent infection	4.76 ± 0.4302	4.66 ± 0.4795	0.3746	− 0.1268–0.3268	0.3866
Practice of food hygiene prevents them from frequent infection	4.6 ± 0.8137	4.66 ± 0.4795	0.7019	− 0.4193–0.2860	0.3866
Clean home and good waste disposal prevent children from frequent infection	4.7 ± 0.4661	4.7 ± 0.4661	> 0.999	− 0.2193–0.2193	0.000
Good respiratory hygiene prevents children from frequent infection	4.5 ± 0.8172	4.7 ± 0.4661	0.4421	− 0.4832–0.2166	0.7793
	46.7 ± 4.027	43.3 ± 6.249	0.0219	0.5284–6.272	2.421

Comparison of the mean score on antibiotics use between group and one-on-one educational intervention is represented in Table 5. Only one item “An antibiotic that was previously prescribed before for a child can be bought again without doctor’s consultation for the same child or another child with similar symptom” had statistically significant difference in mean score of 4.83 ± 0.3790 for the one-on-one education compared to 4.3 ± 1.119 for the group education (*p* = 0.0209). However, the mean score was higher (37.9 ± 3.044) for the one-on-one education compared to 35.93 ± 4.608 for the group education (*p* = 0.0399).

**Table 5:** Comparison of antibiotics use between one-on-one and group after education (*N* = 60)

Variable	One-on-one mean *M* ± SD	Group mean *M* ± SD	*P* value	Confidence Interval	t-score
I ask my doctor or pharmacist to prescribe antibiotics for my child even if they do not want to	4.73 ± 0.4498	4.46 ± 1.042	0.2339	− 0.1820–0.7153	1.216
Antibiotics in powder form can be mixed with hot or warm water before use	4.65 ± 0.8567	4.43 ± 0.9353	0.6102	− 0.3075–0.5144	0.5156
It can be confusing sometimes to know the quantity of water to mix powder antibiotics with	4.7 ± 0.615	4.26 ± 1.112	0.0677	− 0.0336–0.9003	1.898
Measuring the quantity of antibiotic to give a child from powder antibiotic that has been mix can be difficult	4.66 ± 0.7102	4.43 ± 0.9353	0.2566	− 0.1790–0.6457	1.157
Powder antibiotics that has been mixed with water can be stored anywhere	4.76 ± 0.4302	4.6 ± 0.7240	0.1694	− 0.0752–0.4085	1.409
Powder antibiotics that has been mixed with water can be reused after some time either for the same child or another child	4.86 ± 0.357	4.73 ± 0.4498	0.1033	− 0.02878–0.2954	1.682
An antibiotic that was previously prescribed before for a child can be bought again without doctor’s consultation for the same child or another child with similar symptom	4.83 ± 0.3790	4.3 ± 1.119	0.0209	0.0868–0.9798	2.443
Antibiotics can be given at any time outside the recommended dosing time	4.83 ± 0.3790	4.7 ± 0.6513	0.3256	− 0.1394–0.4060	1.000
	37.9 ± 3.044	35.93 ± 4.608	0.0399	0.0972–3.836	2.151

## Discussion

This study sought to evaluate the impact of education on pediatric caregiver’s knowledge of antibiotics and infection prevention and their use of antibiotics. Caregivers’ knowledge of antibiotics and use of antibiotics was generally poor. Participants had better knowledge about infection prevention measures such as hand, food and respiratory hygiene, and immunization in reducing infection in children compared to questions on what antibiotics can treat and their adverse effects, even if both domains improved after education. This could be due to repeated exposure to information on how these measures benefit children and people in general. Pediatric caregiver-focused education is effective at changing parent attitudes toward the use of antibiotics as shown in our study. A study conducted in Malaysia reveals that health education session on appropriate use of antibiotics improved mothers’ level of awareness, practice and attitude about proper use of antibiotic of their children compared to baseline measures [15]. Similarly, in an intervention study using educational videotape on antibiotic use among parents in USA, the parents who were exposed to the videotape were significantly less inclined to seek antibiotics for viral infections than those not exposed [16].

The wrong impression about antibiotics as a one-drug-fit all infections with good safety usually leads to inappropriate antibiotic use, as seen in this study, about half of the participants say that they will buy the same antibiotic previously prescribed for their child again when the child falls sick on their own without a prescription, and one-third of them usually ask their doctors or pharmacists to prescribe antibiotics for their child even if they do not need it. Similar responses have been reported in Eastern Nigeria [17, 18]. Poor knowledge on antibiotics can further encourage unwarranted antibiotic prescribing and overprescribing by physicians due to parental pressure on demands. Physicians who refuse such influence might risk losing those patients to other healthcare facilities or drug store why they can easily get their demands satisfied. This is especially true in settings like Nigeria, where sales of antibiotics are not regulated [19].

Participants in our study seem to understand pediatric antibiotic suspension reconstitution procedure, but with regards to the type of water to reconstitute dry antibiotic with, more than half of the caregivers (71.7%) agree that hot or warm water can be used to reconstitute antibiotics. This practice can reduce the efficacy of antibiotics as high temperature water can decrease the active ingredient compared to those prepared with cooled water at 25 °C of water [20]. In other to avoid reconstitution errors, the reconstitution of medication is done by pharmacists in some developed countries, such as the USA. However, it is done by patients or parents in many low–middle-income countries like Nigeria, because there is limited manpower in the hospital pharmacies. Besides, some instance where the pediatric antibiotic is more than a bottle, it will be unreasonable for a pharmacist to reconstitute all bottles at the same time considering the challenge of lack of constant electricity to properly store the medication in the refrigerator, which some caregivers may not have at home. Therefore, educating parents/caregivers about how to prepare liquid medications from powders properly and administer drugs accurately is crucial to the efficacy and safety of medication in pediatric patients. Pharmacists can begin to include the importance of this important step in writing in addition to verbal instructions [13].

Regarding the educational approach adopted in this study, both the one-on-one interaction and group were effective as the total mean sores increased after education. However, the one-on-one education proved to be more effective in correcting wrong impressions about antibiotics and inappropriate use among pediatric caregivers compared to the group education. The one-on-one education mimics pharmacists’ counseling session with patients in clinical settings. Pharmacists can use the avenue to effectively communicate important details on antibiotics knowledge and proper use to pediatric caregivers to reduce inappropriate use of antibiotics. In a study aimed at improving prescribing behavior of physicians, the authors used one-to-one education and group educational approaches. The one-to-one education obtained an average prescribing behavior improvement of 6.5% in the study period after intervention, whereas in the education group, the average improvement was 2.4% for the same periods [21]. Some advantages of one-on-one education over group education includes a close behavioral observation of patients or participants, such that the health educator can easily adjust what is taught to the participant’s need and level of understanding. This method is termed the most powerful way to influence behavior as it can present opportunities for active learning and patients are more likely to ask questions with this method compared to group learning [22].

The strength of this study lies in the fact that participants are from pediatric caregivers who have used pediatric antibiotic dosage forms and thus provide responses based on their experiences and perceptions. Many uncontrollable factors, like environmental (e.g., noise made by children or movement of people around and non-medical staff calling names of patients) or personal (e.g., impatience or absence of mind, and rush to see the doctor or leave the hospital) may decrease the efficacy of the educational interventions on pediatric caregivers, these are unavoidable limitations.

## Conclusion

This study reveals that pediatric caregivers have poor knowledge about antibiotics and their use but better knowledge on infection prevention measures. Their poor knowledge results in inappropriate antibiotic use and practice. Group and one-on-one education are highly effective in changing wrong perception and practice of antibiotic as shown in our study. One-on-one education proved to be more effective in influencing attitudinal and behavioral change than group education among pediatric caregivers. The one-on-one education mimics pharmacists’ counseling session with patients in clinical settings, pharmacists can use this avenue to effectively communicate important details on antibiotic knowledge and proper use to pediatric caregivers and by extension adult patients to reduce public indiscriminate use of antibiotics.

## Supplementary Information

**Additional file 1.** Data entry for pre and post education.

## Data Availability

All data generated and/or analyzed during the current study are included in this published article.

## Abbreviations

AMR: Antimicrobial resistance
BCIs: Behavioral change interventions
